# Regulation of the Homeostasis of Early Embryo Development in Dairy Cows by Targeted Editing of the *PRLR* Gene-Mediated Activation of the Anti-Heat Stress Pathway

**DOI:** 10.3390/cells14231856

**Published:** 2025-11-25

**Authors:** Xin Cheng, Daqing Wang, Xingyu Zhang, Lu Li, Yiyi Liu, Guifang Cao, Yong Zhang

**Affiliations:** 1 College of Veterinary Medicine, Inner Mongolia Agricultural University, Hohhot 010011, China; 17822106812@163.com (X.C.); wangdaqing050789@126.com (D.W.); 17725760839@163.com (X.Z.); yunsong-5410@163.com (L.L.); 15104718293@139.com (Y.L.); 2 Animal Embryo and Developmental Engineering Key Laboratory of Higher Education, Institutions of Inner Mongolia Autonomous Region, Hohhot 010011, China; 3 Inner Mongolia Autonomous Region Key Laboratory of Basic Veterinary Medicine, Hohhot 010011, China; 4 College of Life Sciences, Inner Mongolia Agricultural University, Hohhot 010011, China; 5 College of Life Sciences, Inner Mongolia University, Hohhot 010021, China

**Keywords:** thermal stress, *PRLR*, gene editing, differentially expressed genes, key genes, somatic cell nuclear transfer

## Abstract

The intensification of global climate warming exacerbates the issue of heat stress in dairy cows, making the *SLICK* mutation in the prolactin receptor (*PRLR*) gene a critical target for enhancing heat tolerance in these animals. This study aims to investigate the effects of CRISPR/Cas9-mediated editing of the *PRLR* gene on the biological characteristics of bovine fibroblasts and early embryonic development following somatic cell nuclear transfer (SCNT). Using the CRISPR/Cas9 system, we targeted and edited a 20 bp–150 bp region within exon nine of the *PRLR* gene. After conducting off-target predictions and activity screenings, we identified optimal guide RNA (sgRNA) sequences and established stable transgenic cell lines. Transcriptome sequencing was performed on edited cells to identify key genes and validate their expression profiles. Edited cells were utilized as donor cells for SCNT, during which we assessed oocyte levels of reactive oxygen species (ROS), glutathione (GSH), and mitochondrial function to analyze embryonic developmental performance. We constructed a cellular stress resistance network aimed at mitigating damage transmission while maintaining embryonic developmental homeostasis. This research provides technical support and theoretical reference for genetic editing breeding programs aimed at improving heat tolerance in dairy cattle.

## 1. Introduction

According to the Intergovernmental Panel on Climate Change (IPCC, 2018), global mean surface temperature is projected to rise by 1.5 °C between 2030 and 2052. The intensification of global warming has increasingly exacerbated the detrimental effects of heat on milk yield, reproductive performance, and immune function in dairy cattle [1]. Prolonged exposure to high ambient temperatures aggravates heat stress, resulting in severe morbidity and mortality, ultimately constraining the sustainable development of the global dairy industry [2].

Among the long-term genetic strategies to mitigate heat stress in dairy cattle, the *SLICK* mutation in the prolactin receptor (*PRLR*) gene has attracted considerable attention. This mutation confers a short, fine hair coat phenotype, enhancing thermoregulation and maintaining high milk yield even under hot conditions, thereby effectively alleviating the inhibitory effects of heat stress on lactation [3]. The slick1 variant, located in exon 10 of the *PRLR* gene, arises from a single cysteine deletion that induces a frameshift mutation, leading to premature translation termination and production of a truncated *PRLR* protein [4]. In contrast, the molecular mechanisms underlying other variants, such as slick2-slick6, remain obscure [5]. Introducing the *SLICK* mutation into the bovine *PRLR* gene via gene-editing technologies has thus emerged as a promising approach to enhancing thermotolerance and ensuring production efficiency of dairy cattle in hot climates [6].

Over the past two decades, the development and refinement of gene-editing tools have provided critical technological support for advancing genetic improvement in livestock. Among these tools, the clustered regularly interspaced short palindromic repeats-associated nuclease 9 (CRISPR/Cas9) system represents a powerful and widely adopted platform. Mechanistically, CRISPR/Cas9 operates through a single-guide RNA (sgRNA) that precisely pairs with a specific target DNA sequence, directing the Cas9 nuclease to induce a double-strand break (DSB) at the target locus [7]. Subsequent repair of the DSB via the error-prone non-homologous end joining (NHEJ) pathway often results in small nucleotide insertions or deletions (indels), which can disrupt gene function by introducing premature stop codons or disrupting protein synthesis, thereby enabling targeted and efficient regulation of gene expression [8].

In the integrated application of gene editing and reproductive biotechnology, somatic cell nuclear transfer (SCNT) plays a pivotal role in enhancing livestock production efficiency and accelerating genetic gain [9]. Prior to nuclear transfer, donor somatic cells can be genetically modified using tools such as CRISPR/Cas9 to introduce targeted gene edits or transgenes, thus supporting the generation of animals with desirable traits [10]. SCNT has become a preferred strategy for achieving multi-gene editing in livestock species. Vazquez-Avendaño et al. (2022) further confirmed its effectiveness and reliability in the production of genetically engineered livestock [11].

Based on this, this study employed the CRISPR/Cas9 system to edit the 20–150 bp region of exon 9 in the bovine *PRLR* gene. Multiple sgRNAs were designed. Through off-target prediction and activity screening, the most effective sgRNA was selected to establish a stably transfected fibroblast cell line. Transcriptomic sequencing of the edited cells was subsequently performed to identify key genes and validate their expression pattern. The edited fibroblasts were then used as donor cells for SCNT. The resulting early embryonic developmental rates were analyzed. Additionally, reactive oxygen species (ROS) levels, glutathione (GSH) content, and mitochondrial function of oocytes post-nuclear transfer were assessed.

This study aimed to systematically explore the biological characteristics of *PRLR*-edited bovine fibroblasts and their influence on embryonic development following SCNT. The findings provide theoretical insights into the mechanistic roles of gene-editing technologies in somatic cell cloning and establish a scientific foundation for breeding thermotolerant dairy cattle adapted to global climate warming, thereby contributing to the sustainable development of the dairy industry.

## 2. Materials and Methods

### 2.1. Oocytes for Experiments

Adult high-quality female dairy cows were selected from the Beiya Youth Holstein Dairy Farm located in Hohhot City, Inner Mongolia Autonomous Region. After slaughter, fresh adult cow ovaries were collected and selected, light red to dark red, shiny oval, smooth surface, length of about 2–4 cm, width of about 1.5–3 cm, thickness of about 1–2 cm, and high-quality bovine oocytes.

### 2.2. Materials and Instruments

IVM medium (IVM, Stroebech Medi, Copenhagen, Denmark) and IVC medium (IVC, Stroebech Medi, Copenhagen, Denmark) were used for in vitro maturation and embryo culture, respectively. Chemicals and reagents included ionomycin calcium salt (I0634, Sigma-Aldrich, St. Louis, MO, USA), 6-(dimethylamino)purine (6-DMAP, D2629, Sigma-Aldrich, St. Louis, MO, USA), and hyaluronidase, Type IV-S (H3884, Sigma-Aldrich, St. Louis, MO, USA), which were employed for cell activation and cumulus cell dispersion. Dulbecco’s phosphate-buffered saline without Ca^2+^/Mg^2+^ (DPBS, 14190-144, Gibco, New York, NY, USA), fetal bovine serum (FBS, 10099-141, Gibco, New York, NY, USA), 0.25% trypsin-EDTA (25200-056, Gibco, New York, NY, USA), DMEM/F-12 supplemented with HEPES (11320-033, Gibco, New York, NY, USA), penicillin-streptomycin (15140-122, Gibco, New York, NY, USA), and Opti-MEM I Reduced-Serum Medium (31985-070, Gibco, New York, NY, USA) were all sourced from Gibco and utilized for cell dissociation, culture, and maintenance of physiological conditions. DH5α competent cells (9057, TAKARA, Shiga, Japan) were used for plasmid propagation. Enzymatic digestion was performed using T7 endonuclease I (T7EI, TransGen Biotech, Beijing, China) and Bbs I restriction endonuclease (R0539S, New England Biolabs, Ipswich, MA, USA). Reactive oxygen species (ROS) levels were assessed using the DCFH-DA assay kit (S0033S, Beyotime, Shanghai, China), and mitochondrial membrane potential was evaluated with MitoTracker Red CMXRos (C1049, Beyotime, Shanghai, China). CellTracker Blue CMF2HC (HY-D0938, MedChemExpress, NJ, USA) was applied for live-cell labeling. Plasmid DNA was purified using the E.Z.N.A. Plasmid Mini Kit I (D6943-01, Omega Bio-Tek, Norcross, GA, USA). The pSpCas9(BB)-2A-EGFP plasmid (Addgene #48138, Addgene, Cambridge, MA, USA) was obtained for CRISPR/Cas9-mediated gene editing applications. Key instrumentation comprised an electrophoresis apparatus (PowerPac Basic, Cat. #1645050, Bio-Rad, Hercules, CA, USA), a gel imaging system (Gel Doc XR+, Cat. #1708195, Bio-Rad, Hercules, CA, USA), a CO_2_ incubator (Heracell™ 240i, Cat. #51032880, Thermo Scientific, Waltham, MA, USA), a confocal laser scanning microscope (LSM 700, Carl Zeiss, Jena, Germany), and a flow cytometer (MA900, SONY, Tokyo, Japan), all of which were calibrated and routinely maintained to ensure experimental accuracy and reproducibility.

### 2.3. Cell Culture

#### 2.3.1. Primary Culture of Fetal Bovine Fibroblasts

Select 4-month-old bovine fetuses. Aseptically excise the ear tissue using sterile scissors and disinfect the tissue by immersion in 75% ethanol for 1–2 min, followed by two washes with DPBS containing 1% penicillin-streptomycin (PS). Mince the tissue into fragments of approximately 1–2 mm^3^ and transfer them into cell culture flasks. Allow the tissue fragments to adhere to the flask surface under standard culture conditions (37 °C, 5% CO_2_) for 5 h. After the attachment period, carefully add complete growth medium supplemented with 10% fetal bovine serum (FBS). Replace the medium every 2–3 days and continue culturing for up to 10 days. Monitor cellular morphology daily under an inverted microscope.

#### 2.3.2. Subculture

When fibroblast confluence reaches 80–90%, aspirate the spent culture medium. Rinse the monolayer gently with DPBS, then add 0.25% trypsin-EDTA solution and incubate at 37 °C for 2 min to dissociate the cells. Neutralize the enzymatic reaction by adding complete culture medium. Transfer the cell suspension to a centrifuge tube and centrifuge at 1000 rpm for 5 min. Aspirate the supernatant, resuspend the cell pellet in fresh medium, and subculture the cells into new flasks or prepare for cryopreservation under standard incubation conditions (37 °C, 5% CO_2_).

#### 2.3.3. Cell Growth Curve Drawing

Fetal bovine fibroblasts of the second and third generations were inoculated into 24-well plates. Every 24 h, the cells were counted using a hematopoietic plate. Three Wells were selected each time for measurement and the average value was taken. This process was continued for 7 days. The obtained data were plotted into a cell growth curve with time (d) as the abscissa and the number of cells as the ordinate.

#### 2.3.4. Cell Chromosome Karyotype Analysis

Cells were selected and transferred to 6-well cell culture plates. After adhering, they were continued to be cultured in culture medium containing 0.2 μg·mL^−1^ colchicine for 5 h. Then, the cells were digested and isolated with trypsin. Subsequently, they were treated with 0.075 mol·L^−1^ KCl hypotonic solution at room temperature for 40 min of hypotonic treatment and fixed with fixative for 8 min. Centrifuge at 1600 r/min for 5 min and repeat 3 times. The cell suspension was dropped onto a cold and clean slide, left to dry at room temperature for 24 h, then stained with Giemsa for 20 min, rinsed under running water, dried naturally, and finally observed under an oil immersion microscope for the changes in chromosome quantity and morphology.

### 2.4. Design of sgRNA, Off-Target Efficiency Detection and Construction of Eukaryotic Expression Vector

#### 2.4.1. sgRNA Design

The exon 9 sequence of the bovine *PRLR* gene (Gene ID: 281422) was selected as the target region for genome editing. Using the CRISPOR online platform “http://crispor.tefor.net (accessed on 8 October 2024)”, potential sgRNA sequences were screened according to the 18–20 nucleotide spacer followed by an NGG protospacer adjacent motif (PAM) rule. Three candidate sgRNAs (sgRNA1, sgRNA2, and sgRNA3) were designed based on this criterion.

#### 2.4.2. Off-Target Efficiency Assessment

In this study, the CRISPR/Cas9 target prediction tool CCTop “https://cctop.cos.uni-heidelberg.de:8043/ (accessed on 13 December 2024)” was employed to evaluate potential off-target effects. CCTop enables the identification and ranking of all candidate sgRNA target sites by assessing their off-target risk profiles. The designed sgRNA sequences were input into the platform, and the corresponding bovine genome was selected for analysis. This allowed comprehensive evaluation of on-target cleavage efficiency and potential off-target binding sites, providing critical information for downstream experimental validation.

#### 2.4.3. Double-Stranded Synthesis of Guide RNA

The pSpCas9(BB)-2A-Puro (PX458, plasmid #48138) vector was selected as the expression vector for targeted genome editing. The oligonucleotide sequences corresponding to the target site were designed (Table 1) and subsequently synthesized by Beijing Qingke Biotechnology Co., Ltd. (Beijing, China).

The synthesized forward and reverse oligonucleotides were each diluted to a final concentration of 100 μM. To generate double-stranded sgRNA with compatible ends for BbsI restriction enzyme cloning, 5 μL of the forward oligo and 5 μL of the reverse oligo were annealed in a PCR thermocycler under the following conditions: initial denaturation at 95 °C for 5 min, followed by a gradual cooling from 95 °C to 22 °C at a rate of 1.5 °C per minute over approximately 50 min. The resulting product was stored at 4 °C until further use.

#### 2.4.4. Construction of U6-sgRNA Plasmid Vector

The PX458 vector was digested with the restriction endonuclease BbsI and linearized by incubation in a 37 °C water bath for 60 min. Gel extraction was performed using a standard agarose gel DNA recovery kit. The digestion reaction mixture consisted of 1 μL of BbsI enzyme (10 U), 1 μg of recombinant PX458 plasmid vector, 5 μL of 10× CutSmart Buffer, and nuclease-free water adjusted to a final volume of 50 μL. The double-stranded sgRNA oligonucleotides were ligated into the linearized PX458 vector at room temperature overnight. The ligation reaction contained 1 μL of T4 DNA Ligase (5 U/μL), 1 μL of 10× T4 DNA Ligase Buffer, 100 ng of linearized PX458 vector, 1 μL of double-stranded sgRNA, and nuclease-free water supplemented to a final volume of 10 μL. Following ligation, the sgRNA sequence was inserted downstream of the U6 promoter in the CRISPR/Cas9 vector, resulting in the construction of pSpCas9(BB)-2A-sgRNA-EGFP.

#### 2.4.5. Plasmid Transformation, Amplification, and Sequencing

All ligation products were transformed into competent DH5α cells via heat shock, and the transformed cells were plated onto LB agar plates containing ampicillin. Single colonies were selected for liquid culture and grown in shaking incubators for plasmid amplification. Plasmid DNA was isolated and submitted for Sanger sequencing to confirm correct insertion. Upon sequence verification, purified recombinant expression vectors—designated PX458-sgRNA1, PX458-sgRNA2, and PX458-sgRNA3—were obtained. Successfully constructed eukaryotic expression vectors were scaled up for large-scale culture. Plasmid DNA from clones with confirmed sequences was extracted using an endotoxin-free plasmid miniprep or maxiprep kit. After quantification via spectrophotometry, the purified plasmids were stored at −20 °C for downstream applications.

### 2.5. Transfection and Activity Detection of Four sgRNA-Expressing Cells

#### 2.5.1. Cell and Plasmid Transfection

Fluorescent expression vectors (PX458-sgRNA1, PX458-sgRNA2, PX458-sgRNA3) were constructed and used for transfection. When the confluence of fetal bovine fibroblasts cultured in 6-well plates reached 70–80%, cells were harvested by trypsinization. The cell suspension was washed twice with OPTI-MEM medium, which served as the electroporation buffer. After resuspension in OPTI-MEM, cells were thoroughly mixed with plasmid DNA. Electroporation was performed using an electroporation system under the following parameters: 175 V, 10 ms pulse duration, single pulse. Post-transfection, cells were transferred to a culture incubator maintained at 37 °C with 5% CO_2_. At 48 h post-transfection, transfected cells were examined under fluorescence microscopy and subsequently cryopreserved for long-term storage.

#### 2.5.2. Detection of sgRNA Cleavage Activity

To assess the cleavage activity of the Cas9 protein directed by sgRNA at endogenous genomic loci, the functional efficiency of the PX458-sgRNA recombinant plasmids was evaluated using the T7EI mismatch cleavage assay.

Cells were collected and resuspended, followed by genomic DNA extraction. The target regions were amplified via PCR using the extracted DNA as template. PCR products were analyzed by 1% agarose gel electrophoresis, and the specific bands were excised and purified. Purified fragments were then subjected to digestion with T7 Endonuclease I (T7EI) at 37 °C for 30 min (Table 2). The digestion products were resolved on a 2% agarose gel to detect cleavage events. The T7EI reaction system consisted of: nuclease-free H_2_O to a final volume of 20 μL, NEB Buffer (2 μL), purified PCR product (10 μL), and T7EI Nuclease (0.2 μL).

### 2.6. Cell Screening and Gene Expression Analysis

#### 2.6.1. Cell Screening

The transfected cells were revived and subsequently re-cultured for 72 h before undergoing fluorescence-activated cell sorting (FACS). Initially, cell aggregates and small debris were excluded by setting gates based on forward scatter area (FSC-A) and side scatter area (SSC-A) parameters. Subsequently, singlet cells were identified using FSC-A versus forward scatter height (FSC-H) to establish a single-cell gate. Positive cells were then selected based on fluorescence signals detected in the FITC-A channel relative to FSC-H. Following FACS, positive cells were seeded into 96-well plates at a density of one cell per well to enable monoclonal expansion. Once confluent, the clones were progressively transferred to larger culture vessels for continued propagation.

#### 2.6.2. Gene Expression Analysis of Stable Cell Lines

After monoclonal cells expanded to confluence in 12-well plates, they were harvested for genomic DNA extraction using a DNA extraction kit. The presence and integrity of the integrated gene construct were verified by PCR amplification with sequence-specific primers (Table 3). Insertion or deletion mutations were further analyzed by Sanger sequencing.

### 2.7. Transcriptome Sequencing and Analysis

#### 2.7.1. Transcriptome Sequencing

Transcriptome sequencing was conducted on cloned cells containing the selected sgRNA. Once the cells reached optimal confluence, they were lysed and shipped to Sangon Biotech Co., Ltd.(Shanghai, China). on dry ice for RNA sequencing. Transcriptomic data were analyzed to compare differential gene expression profiles and pathway enrichment between unedited fibroblasts and genome-edited clone cells.

#### 2.7.2. RT-qPCR Analysis for Validation of Differentially Expressed Genes

To validate the transcriptome sequencing results, selected marker genes were subjected to quantitative real-time PCR (qPCR) using *GAPDH* as an internal reference gene (Table 4). Edited clone cells served as the experimental group, while unedited fibroblasts were used as the control. Each group included three biological replicates, and the experiment was independently repeated three times. Total RNA was extracted from both experimental and control group samples using TRIzol reagent. RNA was subsequently reverse transcribed into cDNA following the instructions for the reverse transcription kit. Quantitative PCR was performed using Takara qPCR kits, the specific steps refer to the instructions.

### 2.8. In Vitro Nuclear Transfer and Cell Biological Assessment of Early Oocyte-Derived Embryos

#### 2.8.1. Somatic Cell Nuclear Transfer (SCNT)

Fresh bovine ovaries were collected, and cumulus-oocyte complexes (COCs) were obtained by aspiration with a 20 mL syringe and placed in a 4-well plate. The COCs were cultured in an incubator at 38.5 °C and 5% CO_2_ for 22 h. Subsequently, the cumulus cells were removed, and the mature oocytes with the second polar body were picked out. The polar body and the surrounding cytoplasm were aspirated by a micromanipulator and injected into the donor nucleus, and the reconstructed embryos were formed by electrofusion. The reconstructed embryos were first activated in ionomycin for 5 min, then transferred to 6-DAMP for 3 h, and then moved to the development fluid. The reconstructed embryos were continuously cultured in incubators at 38.5 °C and 5% CO_2_ for 7 days, and the early embryonic development rate was observed and recorded.

#### 2.8.2. Assessment of Reactive Oxygen Species (ROS), Glutathione (GSH) Levels, and Mitochondrial Distribution in Oocytes

To evaluate the oxidative stress status and mitochondrial characteristics of oocytes following SCNT, two experimental groups were established using different donor cell types. Mature oocytes from each group were denuded and washed twice with phosphate-buffered saline (PBS). Subsequently, oocytes were incubated with specific fluorescent probes: ROS levels were assessed using CellTracker Blue CMF2HC (4-chloromethyl-6,8-difluoro-7-hydroxycoumarin, Invitrogen); intracellular GSH was detected using 2′,7′-dichlorodihydrofluorescein diacetate (H2DCFDA); and mitochondrial localization and integrity were evaluated using MitoTracker Red CMXRos. All staining procedures were conducted at 37 °C for 30 min in the dark. After three washes in PBS, oocytes were placed in glass-bottom dishes containing droplets of PBS and immediately imaged using a Zeiss LSM 700 confocal laser scanning microscope (Carl Zeiss, Oberkochen, Baden-Wurttemberg, Germany). Fluorescence signals were analyzed to assess redox status and mitochondrial function.

## 3. Statistical Analysis

All data and graphical representations were analyzed statistically using GraphPad Prism 9.3.1 software (GraphPad Software, Inc., San Diego, CA, USA) and ImageJ(1.8.0). The mRNA expression levels of target genes were relatively quantified based on fluorescence quantitative PCR results using the 2^−ΔΔCt^ method. Subsequent statistical analyses were performed using SPSS 23.0 (IBM Corp., Armonk, NY, USA), in which independent samples t-tests were employed to assess the significance of differences in gene expression. All data had been analyzed with the mean ± standard deviation (SD). Statistical significance was evaluated by one-way analysis of variance (ANOVA) followed by Tukey’s multiple comparisons test or two-way ANOVA (Bonferroni’s post-test), as appropriate. Differences with *p* values ≤ 0.05 were considered statistically significant(ns *p* > 0.05, * *p* < 0.05, ** *p* < 0.01 compared to the normal group).

Agarose gel electrophoresis was performed on the reaction products of T7EI, and the gray values of the electrophoresis images were calculated using Image J to obtain the cleavage efficiencies of different sgRNAs. Cutting efficiency standards are as follows (Table 5).

## 4. Results

### 4.1. Cell Culture and Identification

**Figure 1:**
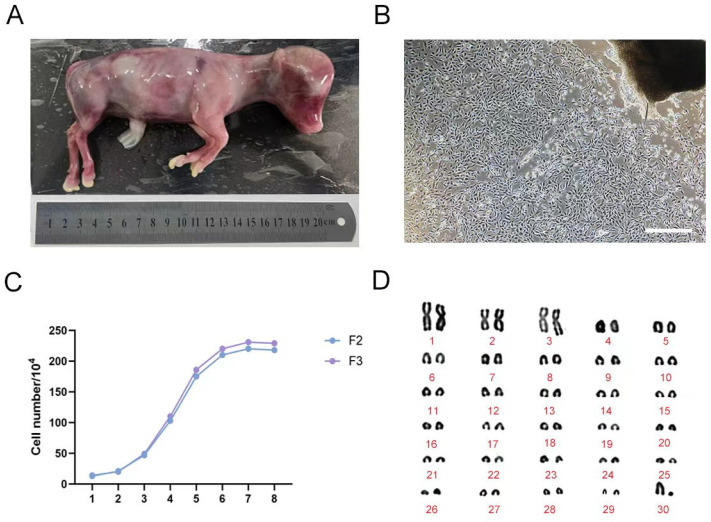
Culture and identification of fetal bovine fibroblasts. (**A**) 4-month-old fetal cattle; (**B**) Primary fetal bovine fibroblast morphology (250 μm); (**C**) The growth curve of fetal bovine F2, F3 fibroblasts; (**D**) Chromosome karyotype of F3 fetal bovine fibroblasts.

Fetal calves at a gestational age of four months were obtained for the study. The fetuses measured approximately 20 cm in length and exhibited a smooth, hairless appearance, consistent with typical morphological characteristics of bovine fetuses at this developmental stage (Figure 1A). Primary fibroblast cultures were established using marginal ear tissues from the fetal bovine specimens. After ten days of adherent tissue block culture, the majority of outgrown cells displayed a long spindle-shaped or irregular triangular morphology, with oval nuclei, clearly visible nucleoli, and robust cellular growth (Figure 1B), indicating successful isolation and cultivation of fetal bovine ear-derived fibroblasts.

Growth curves were generated for F2 and F3 generation fibroblasts (Figure 1C). The data revealed a classical growth pattern consisting of three distinct phases: a lag phase during days 1–3, followed by a logarithmic phase of rapid proliferation from days 3 to 6, and culminating in a plateau phase of slowed growth between days 6 and 8—collectively exhibiting an “S”-shaped growth curve. Additionally, karyotype analysis was performed on the cultured fibroblasts (Figure 1D), confirming a diploid chromosome number of 30 pairs (2n = 60), with no observable chromosomal abnormalities. This karyotypic profile is consistent with the expected chromosomal complement for Bos taurus.

### 4.2. Design and Construction of Eukaryotic Expression Vector of sgRNA

#### 4.2.1. Vector Construction

**Figure 2:**
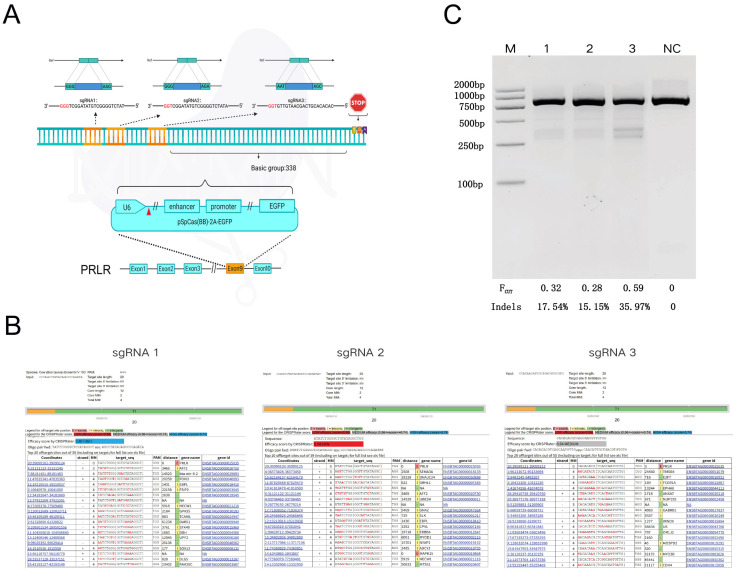
sgRNA Design and Evaluation of *PRLR* Gene Editing Efficiency, Off-Target Analysis, and sgRNA Activity Assessment. (**A**): Oligonucleotides targeting exon 8 of the *PRLR* gene were designed and cloned into a targeted vector to construct sgRNA expression plasmids. Three sgRNAs (sgRNA1, sgRNA2, and sgRNA3) were designed and inserted into the pSpCas9(BB)-2A-EGFP vector under the control of the U6 promoter. The PAM sequences are indicated in red. Gene: *PRLR*; Genomic location: Exon 8; Sequence orientation: 5′→3′. The diagram illustrates that the three sgRNA target sites are located 338 base pairs upstream of the stop codon. (**B**): Potential off-target sites were predicted using the CCTop platform, and sgRNA targeting efficiencies were scored accordingly (sgRNA1: high efficiency; sgRNA2: low efficiency; sgRNA3: medium efficiency). (**C**): T7EI nuclease digestion followed by agarose gel electrophoresis and ImageJ-based densitometric analysis were performed to assess editing efficiency. M: Trans DNA Marker II; NC: PCR product from cells transfected with Cas9 vector lacking sgRNA insertion (890 bp); Lane 1: sgRNA1 target site; Lane 2: sgRNA2 target site; Lane 3: sgRNA3 target site. Fcut: cleavage rate; Indels: actual indel frequency, calculated as the ratio of heteroduplex to total double-stranded DNA.

According to the Cas9 target design principle, three sgRNAs (sgRNA1, sgRNA2, and sgRNA3) targeting distinct sites within exon 9 of the *PRLR* gene in dairy cattle were designed, using PX458 as the backbone vector. The respective sgRNA sequences were inserted downstream of the U6 promoter in the PX458 plasmid through enzymatic digestion, annealing, and ligation (Figure 2A).

#### 4.2.2. Predicting the Potential Off-Target Efficiency

The evaluation of sgRNA off-target efficiency revealed significant differences in both efficiency and off-target risk among the three sgRNAs targeting the *PRLR* gene (Figure 2B). Specifically, sgRNA1 exhibited a CRISPRater score of 0.80, indicating high on-target efficiency, with the off-target site located farthest from the intended target, thus demonstrating the highest specificity. In contrast, sgRNA2 yielded a score of 0.54, reflecting low efficiency, and was associated with short-range off-target effects and a higher risk profile. sgRNA3 achieved a score of 0.64, corresponding to moderate efficiency; although it displayed few close-range off-target sites, the total number of potential off-targets was slightly elevated.

#### 4.2.3. Identification and Analysis of sgRNA Cleavage Activity

The T7EI enzymatic digestion assay revealed a single uncut band in the negative control (NC) group, whereas cells transfected with various sgRNA vector plasmids exhibited distinct cleavage bands, indicating successful genome editing. Densitometric analysis of the bands was performed using ImageJ software, which quantified the cleavage efficiencies of PX458-SgRNA1, PX458-SgRNA2, and PX458-SgRNA3 at 17.54%, 15.15%, and 35.97%, respectively (Figure 2C).

Considering both target site cleavage efficiency and potential off-target effects, sgRNA3 was selected for subsequent experiments to generate *PRLR*-edited fetal bovine fibroblast cell lines.

### 4.3. Screening of Fetal Bovine PRLR Gene Editing Cells and Gene Identification

**Figure 3:**
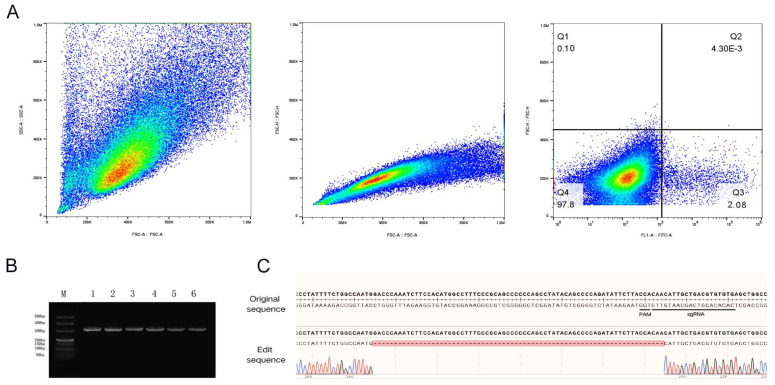
Screening of stably transfected gene-edited cell strains. (**A**): Flow cytometry sorting workflow. The main cell population was identified using FSC-A versus SSC-A parameters; single-cell populations were isolated based on FSC-A and FSC-H correlation; subsequent sorting was performed via FITC-A versus FSC-H, revealing 2.08% positive cells in the Q3 gate. (**B**): Agarose gel (1%) electrophoresis results. Lane M contains DNA molecular weight markers; lanes 1–3 display PCR amplification products from fibroblasts; lanes 4–6 show PCR amplification results from single-cell clones. (**C**): Sanger sequencing chromatogram of genomic DNA from edited cells. The editing site is located within the final three nucleotides preceding the PAM sequence, resulting in a 70 bp deletion.

Following co-transfection of the PX458-sgRNA3 vector plasmid containing the eGFP reporter gene into fetal bovine fibroblasts, fluorescence-activated cell sorting (FACS) was employed for cell screening. Final statistical analysis revealed that the proportion of EGFP-positive monoclonal cells was 2.08% (Figure 3A).

After expansion of the selected monoclonal cell populations, genomic DNA was extracted and subjected to PCR amplification. Agarose gel electrophoresis results (Figure 3B) demonstrated that both the control fragment NC and the PCR products amplified from the sgRNA3 target site migrated to the 330 bp position, indicating successful amplification. Subsequent Sanger sequencing of the PCR products was performed, and the obtained sequences were aligned with the reference sequence of the *PRLR* gene. Analysis confirmed a 70 bp deletion mutation within the targeted region of the *PRLR* gene (Figure 3C). Further quantitative assessment revealed a gene editing efficiency of 29.23% (19 out of 65 clones analyzed).

### 4.4. Transcriptome Results Analysis and Marker Gene Verification

Transcriptome sequencing analysis was performed on fetal bovine fibroblasts from the control group and genome-edited cells in the experimental group to assess differential gene expression through comparative transcriptomic profiling.

**Figure 4:**
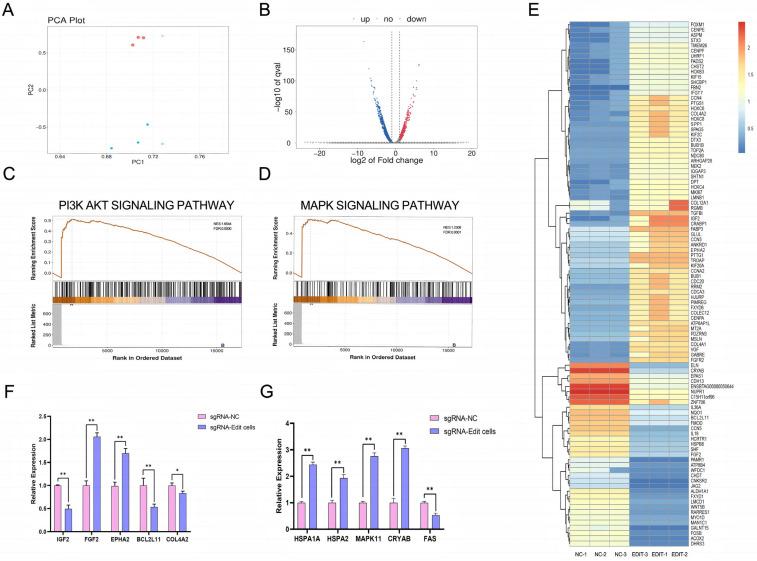
Analysis of the effects of sgRNA editing on cellular gene expression and associated signaling pathways. (**A**): PCA plot illustrating overall differences in gene expression profiles across samples and demonstrating intra-group reproducibility. Different colors denote distinct experimental groups, while the spatial distribution of data points reflects clustering patterns of gene expression profiles. (**B**): Volcano plot depicting differentially expressed genes (DEGs). The *x*-axis represents log_2_(Fold Change), indicating the magnitude of differential expression, while the *y*-axis shows -log_10_(*p* value), reflecting statistical significance. Upregulated, downregulated, and non-significant DEGs are represented by red, blue, and gray dots, respectively. (**C**): Enrichment trend plot for the PI3K-AKT signaling pathway. The upper curve illustrates the enrichment score distribution across the ranked gene list, while the lower curve displays the positions of differentially expressed genes within this pathway. (**D**): Enrichment trend plot for the MAPK signaling pathway. The upper curve indicates the pathway’s enrichment profile, and the lower curve shows the distribution of differentially expressed genes along the ranked gene list. (**E**): Heatmap of differentially expressed genes. Rows correspond to individual DEGs, and columns represent samples. Red and blue shades indicate high and low levels of gene expression, respectively. (**F**): Analysis of relative expression levels of differentially expressed genes associated with the PI3K-AKT signaling pathway(*: *p* < 0.05; **: *p* < 0.01). (**G**): Analysis of relative expression levels of differentially expressed genes associated with the MAPK signaling pathway (**: *p* < 0.01).

Principal component analysis (PCA) revealed a clear separation between sample distributions in the control and experimental groups, with tight clustering within each group (Figure 4A), indicating distinct gene expression patterns between the two groups and high intra-group reproducibility.

Volcano plot analysis for differential gene expression demonstrated substantial transcriptional alterations in edited fibroblasts compared to controls.

Specifically, 1587 genes were significantly upregulated and 2026 genes were significantly downregulated (Figure 4B), suggesting that genome editing induced significant changes in gene expression profiles.

Differential gene expression patterns (heat map): The heatmap illustrates distinct expression profiles of differentially expressed genes between the two sample groups, clearly demonstrating the impact of sgRNA-mediated editing on the cellular transcriptome. Notably, several genes associated with stress response and damage repair were significantly upregulated, including *CRYAB*, *HSPB8*, and *FOSB* (Figure 4E).

Pathway analysis reveals that, compared to the control group, the experimental group exhibits significant enrichment in biological pathways related to stress response, DNA damage repair, and cell cycle regulation. The differentially expressed genes are predominantly enriched in the *PI3K-AKT* and *MAPK* signaling pathways (Figure 4C,D).

Genetic verification: Among the genes associated with the *MAPK* signaling pathway, the experimental group exhibited significantly elevated expression levels of stress-related genes *HSPA1A, HSPA2, MAPK11*, and *CRYAB* compared to the control group, with highly significant differences (*p* < 0.01). In contrast, the expression level of the pro-apoptotic gene FAS was markedly reduced, showing a highly significant difference (*p* < 0.01). Within the *PI3K-AKT* pathway-related genes, the expression levels of FGF2 and EPHA2 were significantly upregulated in the experimental group, whereas those of *IGF2*, *BCL2L11*, and *COL4A2* were downregulated. Notably, the changes in *FGF2*, *EPHA2*, *IGF2*, and *BCL2L11* were highly significant (*p* < 0.01), while the reduction in *COL4A2* was statistically significant (*p* < 0.05) (Figure 4F,G). These findings collectively indicate that the observed differential gene expression between the two groups is statistically robust. The results suggest that cells in the experimental group possess enhanced tolerance to cellular stress, potentially mediated through the activation or modulation of stress defense and cell state regulatory pathways, particularly the *MAPK* and *PI3K-AKT* signaling pathways.

In conclusion, sgRNA-mediated editing substantially alters the cellular gene expression profile, affecting key signaling pathways such as *PI3K-AKT* and *MAPK*, and may thereby regulate diverse biological processes.

### 4.5. Nuclear Transfer and Cell Biology Detection

#### 4.5.1. Oocyte Development and Cleavage Rate Statistics

In this study, fibroblasts (control group) and gene-edited cells (experimental group) were employed as donor cells for somatic cell nuclear transfer, and the reconstructed embryos were cultured in embryo culture medium under in vitro conditions. The results indicated that the cleavage rates were 73% in the control group and 72% in the experimental group, while the blastocyst formation rates were 30% and 27%, respectively (Table 6). Statistical analysis revealed no significant differences in either cleavage rate or blastocyst formation rate between the two groups.

#### 4.5.2. Detection of ROS, GSH and Mitochondria in Oocytes

**Figure 5:**
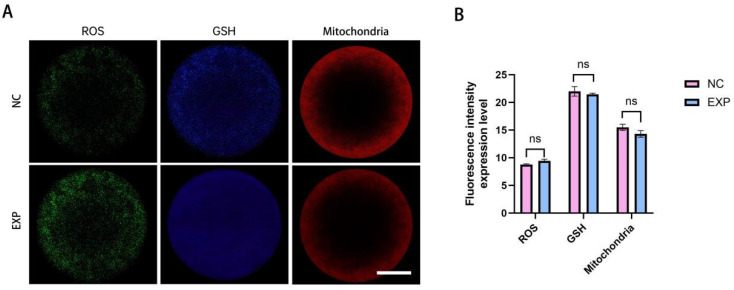
Analysis of oocyte nuclear transfer outcomes following gene editing. (**A**): Representative images depicting ROS, GSH levels, and mitochondrial ultrastructure in oocytes after nuclear transplantation and fusion in both the experimental and control groups. (**B**): Quantitative comparison of ROS, GSH, and mitochondrial fluorescence intensities between the control and experimental groups; no statistically significant differences were observed (*p* > 0.05). (Scale bar: 20 μm).

CMF2HC staining was employed to detect reactive oxygen species (ROS) levels in oocytes. The results showed no significant difference in relative fluorescence intensity of ROS between the experimental group and the control following fusion, indicating no statistically significant alteration in ROS content (*p* > 0.05). Similarly, no significant difference was observed in glutathione (GSH) levels between the groups (*p* > 0.05). Mitochondrial distribution was assessed using MitoTracker Red CMXRos staining. In the experimental group, mitochondria were predominantly localized in the cortical and perinuclear regions of oocytes. The fluorescence signal exhibited a uniform distribution pattern, with no notable differences in intensity compared to the control (*p* > 0.05) (Figure 5A,B).

## 5. Discussion

Global warming has become a profound threat to the sustainability of livestock production, imposing substantial economic burdens on the animal husbandry industry [12]. In tropical and subtropical regions, heat stress represents a primary constraint on livestock productivity, leading not only to significant declines in dairy performance but also, under prolonged heat waves, to large-scale livestock mortality [13,14]. With the intensification of global warming, effective strategies involving nutritional modulation, genetic improvement, and optimization of environmental management have been developed to safeguard animal welfare and sustain productive efficiency [15].

Existing studies have identified the presence of a “slick” mutation in the *PRLR* gene among Criollo cattle populations in South and Central America, conferring a pronounced thermotolerance advantage [16]. This mutation enables more effective thermoregulation and sustains milk production under heat stress. Within the family of SLICK phenotypes, all reported mutation sites are clustered in the downstream coding region of the *PRLR* gene, particularly in exon 10 [17]. *PRLR* gene contains 10 exons. Exon 2–7 encodes the extracellular domain, 8 encodes the transmembrane domain, and 9 and 10 jointly encode the intracellular signal domain. Exon 9 is the upstream part of the intracellular domain. Editing it can directly block the signal of hair follicle development, and its sequence is short and the target is accurate, which does not affect the protein binding and localization. Combined with the current situation that the SLICK phenotype is mostly achieved by editing exon 10 in the existing research, exon 9 can be used as a new target for gene editing of this phenotype. Therefore, this study targeted exon 9 of the *PRLR* gene using CRISPR/Cas9-mediated editing to explore its influence on protein function, cellular thermotolerance, and embryonic development—representing a distinct strategy from previous exon 10-focused approaches. This investigation thus provides novel insights into the potential functional roles of different *PRLR* exons in regulating thermotolerance and offers new target options and mechanistic references for gene-editing-based dairy cattle breeding.

Using the *PRLR* gene as a core target, this study successfully established bovine fetal fibroblast lines carrying a 70 bp deletion mutation in exon 9 through precise CRISPR/Cas9-mediated editing. Transcriptomic analysis and gene validation revealed significant alterations in two key signaling pathways—MAPK and PI3K-AKT—indicating their strong association with cellular stress defense, survival regulation, and the subsequent adaptability of cells for SCNT.

Within the MAPK pathway, *HSPA1A* and *HSPA2,* members of the heat shock protein (HSP) family, were significantly upregulated (*p* < 0.01). These molecular chaperones play pivotal roles in cellular defense against stress. *HSPA2* facilitates protein folding and assembly [18], while the *HSP* family more broadly functions to recognize and bind denatured proteins [19], maintaining proper protein conformation and mitigating aggregation-induced damage during heat stress. The upregulation of *MAPK11* (*p* < 0.01), a key component of the p38 MAPK subfamily, may further activate DNA damage repair pathways, thereby reducing the accumulation of mutations arising from DSBs during gene editing and enhancing genomic stability. *CRYAB* can mitigate stress-induced cytoskeletal aggregation and reduce oxidative damage [20]. Accordingly, in this study, the upregulation of *CRYAB* (*p* < 0.01) was found to not only preserve cytoskeletal integrity but also alleviate oxidative stress-induced damage by scavenging ROS. Concurrently, the significant downregulation of the pro-apoptotic gene *FAS* (*p* < 0.01) further attenuated stress-induced cell death by modulating apoptotic signaling pathways.

Activation of phosphoinositide 3-kinase (PI3K) leads to phosphorylation and activation of Akt, which promotes cell survival by enhancing the expression of anti-apoptotic proteins and suppressing the activity of pro-apoptotic factors [21]. In the PI3K-AKT signaling pathway, the significant upregulation of fibroblast growth factor 2 (*FGF2*) and EPH receptor A2 (*EPHA2*) (*p* < 0.01) suggests enhanced metabolic regulation and anti-apoptotic capacity through downstream activation of AKT kinases. *FGF2* facilitates cell cycle progression and maintains proliferative activity under in vitro culture conditions, while EPHA2 improves cell adhesion properties. Meanwhile, the downregulation of *IGF2*, *BCL2L11*, and *COL4A2* (*IGF2* and *BCL2L11*, *p* < 0.01; *COL4A2*, *p* < 0.05) reflects an adaptive cellular regulatory strategy rather than simple functional inhibition: moderate suppression of *IGF2* reduces metabolic burden, prioritizing resource allocation toward stress defense mechanisms, and downregulation of *BCL2L11* directly weakens apoptotic signal transmission. Peng et al. (2025), using a decellularized extracellular matrix (dECM) model of breast cancer, demonstrated that *COL4A2* overexpression increases ECM stiffness, whereas its silencing reduces ECM elasticity and limits cancer cell invasiveness [22]. Accordingly, the decreased *COL4A2* expression observed in this study may reduce ECM rigidity, thereby providing a more flexible microenvironment conducive to cellular growth.

Rezatabar et al. (2019) confirmed that the RAS/MAPK signaling pathway plays a crucial role in oxidative stress and DNA damage responses [23]. Huang et al. (2024) reported that the interaction between the MAPK and PI3K-AKT pathways effectively mitigates stress-induced damage to cellular integrity and barrier function [24]. These two pathways represent a complementary division of roles: the MAPK pathway primarily focuses on “heat damage mitigation and maintenance of intracellular homeostasis,” while the PI3K-AKT pathway mainly involves “metabolic optimization and enhancement of extracellular adaptation and repair capacity.” Together, they form a comprehensive molecular network conferring thermotolerance at the cellular level. This mechanism aligns with the *PRLR* gene SLICK mutation, which produces a short, sleek hair phenotype advantageous for heat dissipation—illustrating a “molecule-to-phenotype” linkage and further validating the *PRLR* gene as a scientifically robust and functionally central target for improving thermotolerance in dairy cattle.

From the perspective of early embryonic development, this study employed *PRLR*-edited fibroblasts as donor cells for SCNT. The results revealed no significant differences in cleavage and blastocyst rates between reconstructed embryos derived from *PRLR*-edited cells and those from unedited fibroblast donor cells. Similarly, oocyte ROS levels, GSH content, and mitochondrial distribution showed no statistical differences (*p* > 0.05), indicating that embryonic developmental homeostasis was preserved. This outcome can be attributed to the pre-activation of MAPK and PI3K-AKT pathways. Sustained activation of *MAPK11* likely accelerated DNA damage repair and reduced residual DSBs caused by gene editing, thereby maintaining genomic stability. The upregulation of *CRYAB* effectively scavenged ROS within oocytes, protecting both DNA and protein integrity from oxidative damage. Moreover, Du et al. (2021) demonstrated that *FGF2/FGFR* signaling promotes in vitro maturation of cumulus-oocyte complexes [25]. Accordingly, the elevated *FGF2* expression in this study likely contributed to the maintenance of cellular metabolism and proliferation. Concurrently, the downregulation of *BCL2L11* and *FAS* suppressed apoptotic signaling, thereby reducing the risk of oxidative stress-induced cell death. Maidarti et al. (2020) reported cross-regulation between the PI3K-AKT pathway and oocyte DNA damage repair mechanisms, underscoring their crucial role in maintaining oocyte quality [26]. In alignment with this, the coordinated activation of the MAPK and PI3K-Akt pathways minimized stress-induced damage transmission, enabling reconstructed oocytes to maintain redox and developmental homeostasis without activating additional oxidative defense mechanisms.

Nevertheless, this study has certain limitations. The long-term physiological effects of *PRLR* gene editing in cattle remain to be elucidated. As this investigation primarily focused on the cellular level and early embryonic development, further research employing transgenic animal models is warranted to comprehensively assess the heat tolerance, milk production performance, and reproductive capacity of *PRLR*-edited cattle. In particular, the genetic stability and phenotypic heritability of the SLICK allele in Holstein breeds require systematic validation.

Despite these limitations, this study provides direct experimental evidence supporting the feasibility of efficient CRISPR/Cas9-mediated editing of the *PRLR* gene in bovine fetal fibroblasts. Moreover, it clarifies the molecular mechanisms underlying the regulatory effects of gene editing on cellular stress responses and embryonic development, primarily through MAPK and PI3K-Akt signaling coordination. In the context of intensifying climate change and its detrimental impact on livestock production, the precise targeting of thermotolerance-associated genes offers a promising strategy for genetic improvement. This study not only establishes a technical foundation for heat-tolerant cattle breeding but also contributes theoretical insights into the application of gene-editing technology in animal somatic cell cloning. Subsequent research could further address these limitations to advance the industrial implementation of gene editing in bovine genetic improvement and promote the sustainable development of the dairy industry.

## 6. Conclusions

This study aimed to mitigate heat stress in dairy cows through targeted genome editing. By employing CRISPR/Cas9 technology to specifically modify exon 9 of the prolactin receptor (*PRLR*) gene in bovine fetal fibroblasts, a stable cell line harboring a 70 bp deletion mutation was successfully generated. Furthermore, the activation of the MAPK and PI3K-AKT signaling pathways contributed to the formation of a cellular anti-stress network, which effectively attenuated the transmission of heat stress-induced damage. Notably, this genetic intervention did not compromise the early developmental potential of somatic cell nuclear transfer embryos, thereby providing both technical support and a theoretical foundation for gene-editing-based breeding strategies aimed at enhancing thermotolerance in dairy cattle.

## Figures and Tables

**Table 1:** sgRNA sequence and oligonucleotide sequence of corresponding targeting sequence.

sgRNA of Number	sgRNA Sequence (PAM:NGG) (5′-3′)	Guide Oligo (5′-3′)
1	TATCTGGGGCGTATAGGCT (GGG)	Top: CACC TATCTGGGGCGTATAGGCT Bottom: AAAC AGCCTATACGCCCCAGATA
2	ATATCTGGGGCTGTATAGGC (TGG)	Top: CACC ATATCTGGGGCTGTATAGGC Bottom: AAAC GCCTATACAGCCCCAGATAT
3	CACACACGTCAGCAATGTTG (TGG)	Top: CACC CACACACGTCAGCAATGTTG Bottom: AAAC CAACATTGCTGACGTGTGTG

**Table 2:** Primer for T7EI-*PRLR*-amplicon.

Primer Name	Primer Sequence (5′-3′)	Amplicon Length
T7EⅠ-*PRLR*-amplicon	F:GACTGCGAGGACTTGCTGAT	890 bp
	R:ACAGAGTCAGGTTTTGCGCT	

**Table 3:** List of primers for polymerase chain reaction (PCR).

Amplicon Name	Primer Sequence (5′-3′)	Length (bp)
*PRLR*	F:CCACAACATTGCTGACGTGT	400
	R:TACTCCTTGCTGGCTTCAGG	

**Table 4:** Marker Gene Detection Primers.

Amplicon Name	Primer Sequence (5′-3′)	Length (bp)
*GAPDH*	F:ACGGGAAGCTCACTGGCATGG	227 bp
	R:GCCAGCCCCAGCATCGAAG	
*HSPA1A*	F:GTGGAGGATGAGGGGCTGAAG	80 bp
	R:GAAATCACCTCCTGGCACTTGTC	
*HSPA2*	F:ACCTTCGACGTGTCCATCCTG	145 bp
	R:CCTTCTTGTGCTTGCGTTTGAAC	
*MAPK11*	F:TACCTGGTGACCACGCTGATG	137 bp
	R:TGGATGATCCCCGCCGAATG	
*CRYAB*	F:TGATCTCTTCCCAGCTTCTACTTCC	116 bp
	R:TGTCCTTCTCCAGACGCATCTC	
*FGF2*	F:AAGAGCGACCCACACATCAAAC	104 bp
	R:CCATCTTCTTTCATAGCAAGGTAACG	
*EPHA2*	F:TACACGGAGAAGCAGCGAGTAG	114 bp
	R:CATCAGAGGTTTGTACTTGGAGACG	
*IGF2*	F:CTCGTGCTGCTATGCTGCTTAC	113 bp
	R:TGGATGGTCGGCTGAAGTAGAAG	
*BCL2L11*	F:CAACCTTCCGATGTAAGTTCTGAGTG	122 bp
	R:CTCCTGTCTTGCCGCTCTGTC	
*COL4A2*	F:CGGAGAGCCCAACACCCTTC	116 bp
	R:TGCCTGGAAAGCCCTGAAGTC	
*FAS*	F:GAGTACACAGACAAGAGCCATCATTC	82 bp
	R:GTTCCACTTCTAGCCCATGTTCTTC	

**Table 5:** Formula for calculating cutting efficiency.

Name	Formula
Cutting efficiency	Fcut = Sum of gray values of two strips formed by enzyme digestion/sum of gray values of three strips in this lane
The actual cutting efficiency of Cas9 protein at the target site	Indel% = 1 − (1 − Fcut) 1/2 × 100

**Table 6:** Number of early embryo cleavage.

Sample	Number of Somatic Cell Nuclear Transfer Cells (Repeats)	Percentage (Number ± SD)					
		2 Cell	4 Cell	8 Cell	16 Cell	Morula	Blastocyst
Control	300 (3)	73 (219 ± 1.12)	67 (201 ± 2.52)	52 (156 ± 1.83)	43 (128 ± 1.56)	35 (106 ± 1.73)	30 (91 ± 0.67)
Treated	300 (3)	72 (216 ± 3.26)	64 (192 ± 1.12)	49 (147 ± 2.73)	40 (119 ± 0.64)	33 (100 ± 2.44)	27 (82 ± 1.27)

Note: Three independent biological replicate statistics (*n* = 3, mean ± standard deviation).

## Data Availability

The original contributions presented in this study are included in the article. Further inquiries can be directed to the corresponding author.
